# Changes in the criticality of Hopf bifurcations due to certain model reduction techniques in systems with multiple timescales

**DOI:** 10.1186/2190-8567-1-9

**Published:** 2011-09-23

**Authors:** Wenjun Zhang, Vivien Kirk, James Sneyd, Martin Wechselberger

**Affiliations:** 1 Department of Mathematics, University of Auckland, Auckland, New Zealand; 2 School of Mathematics and Statistics, University of Sydney, Camperdown, NSW, Australia

**Keywords:** physiological model reduction, geometric singular perturbation theory, Hopf bifurcation; first Lyapunov coefficient, quasi-steady-state reduction

## Abstract

A major obstacle in the analysis of many physiological models is the issue of model simplification. Various methods have been used for simplifying such models, with one common technique being to eliminate certain 'fast' variables using a quasi-steady-state assumption. In this article, we show when such a physiological model reduction technique in a slow-fast system is mathematically justified. We provide counterexamples showing that this technique can give erroneous results near the onset of oscillatory behaviour which is, practically, the region of most importance in a model. In addition, we show that the singular limit of the first Lyapunov coefficient of a Hopf bifurcation in a slow-fast system is, in general, not equal to the first Lyapunov coefficient of the Hopf bifurcation in the corresponding layer problem, a seemingly counterintuitive result. Consequently, one cannot deduce, in general, the criticality of a Hopf bifurcation in a slow-fast system from the lower-dimensional layer problem.

## 1 Introduction

Many models of physiological processes have the feature that one or more state variables evolve much faster than the other variables. Classic examples are neural activities such as bursting and spiking, and intracellular calcium signalling [1]. In many of these models, the time scale separation becomes apparent in the form of a small dimensionless parameter (often denoted by *ε*) after non-dimensionalisation of the model that brings it into a standard slow-fast form:^a^

(1) $$\begin{array}{c} x^{\prime }=f\left(x,z;\mu ,\varepsilon \right),\\ z^{\prime }=\varepsilon g\left(x,z;\mu ,\varepsilon \right), \end{array}$$

where *x *∈ ℝ*^k ^*denotes the fast (dimensionless) state variables, *z *∈ ℝ*^l ^*denotes the slow (dimensionless) state variables, *μ *∈ ℝ*^m ^*denotes (dimensionless) parameters of the model, prime denotes differentiation with respect to the fast (dimensionless) time scale *t*, and *ε *≪ 1. Such a model has an equivalent representation on the slow time scale *τ *= *εt*, obtained by rescaling time and given by

(2) $$\begin{array}{ll} \varepsilon ẋ & =f\left(x,z;\mu ,\varepsilon \right),\;\\ ż & =g\left(x,z;\mu ,\varepsilon \right),\;\\ & \; \end{array}$$

where the overdot denotes differentiation with respect to the slow time scale *τ*. Models with this feature are called singularly perturbed systems and one can exploit the separation of time scales in the analysis of these (*k *+ *l*)-dimensional models by splitting the system into the *k*-dimensional fast subsystem obtained in the singular limit *ε *→ 0 of (1) and known as the *layer problem*, and the one-dimensional slow subsystem obtained in the singular limit *ε *→ 0 of (2) and known as the *reduced problem*. The aim is to make predictions about the dynamics in the full model based on what is seen in the lower-dimensional fast and slow subsystems. Geometric singular perturbation theory (GSPT) [2-9] forms the mathematical foundation behind this approach and it is a well-established tool in the analysis of many multiple time scales problems in the biosciences (see, e.g., [1,10-12]).

Perhaps the best-known instance of the use of GSPT in this way is the analysis of the famous Hodgkin-Huxley (HH) model of the (space-clamped) squid giant axon [13] by FitzHugh [14,15]. The HH model is a four-dimensional conductance-based model in which two state variables (the inactivation gate of the sodium channel *h *and the activation gate of the potassium channel *n*) have slow kinetics compared to the other two fast state variables (the membrane potential *V *and the activation gate of the sodium channel *m*). Thus, it is possible to split the analysis of this four-dimensional problem into a two-dimensional layer problem and a two-dimensional reduced problem which are amenable to phase-plane analysis.

Concatenation of solutions of these two subsystems then allows an explanation of the genesis of, e.g., action potentials observed in the full model.

FitzHugh [15] and Nagumo [16] introduced a Van der Pol-type two-dimensional model reduction (the now famous FHN model) which captures the essential qualitative dynamics of the HH model. Rinzel [17,18] then performed a physiological model reduction of the HH equations to a model with one slow (*n*) and one fast (*V *) state variable that also retained the qualitative behaviour of the neural dynamics observed. Rinzel's first reduction step is to relax the fast gate *m *instantaneously to its quasi-steady-state value *m *= *m*_∞_(*V*). This model reduction 'technique' of relaxing fast gates to their quasi-steady-states is used in many conductance-based models. In this article, we will show when such a reduction step is mathematically justified and point out some potential problems of this technique.

The second reduction step used by Rinzel is based on a numerical observation about the dynamics of the slow variables, namely that there seems to be a (linear) functional relation along the attractor between *n *and *h *such that one can replace *n *by a function of *h *(FitzHugh [15] observed this as well). This step has no mathematical justification but the two-dimensional model obtained in this way still describes the basic HH model dynamics well. Of course, some transient features of the original model are lost [19] as well as possible chaotic behaviour [20,21]. These transient features might become important when one models coupled cells where such intrinsic transient dynamics might play a role in forming new attractors.

In many physiological models, we are interested in the onset of oscillations, i.e. in the existence and criticality of Hopf bifurcations. The existence and location of any Hopf bifurcations in a model can easily be established by computing the eigenvalues of the system linearised about the equilibrium solutions; a Hopf bifurcation occurs generically when a pair of eigenvalues crosses the imaginary axis under parameter variation. However, determination of the criticality of a Hopf bifurcation typically is more complicated. For a general system, criticality of a Hopf bifurcation is computed using centre manifold theory to reduce the problem to a two-dimensional system, valid near the Hopf bifurcation, and then doing calculations on the model restricted to this two-dimensional centre manifold. These calculations determine the so-called first Lyapunov coefficient for the Hopf bifurcation [22,23], the sign of which determines whether or not the Hopf bifurcation is supercritical, i.e. which side of the Hopf bifurcation the oscillations appear and whether they are stable on the centre manifold. It is desirable that model reductions be performed in such a way that a Hopf bifurcation in the full model corresponds to a Hopf bifurcation in the reduced model and that the criticalities of the bifurcations in the full and reduced models match. We will point out where model reductions may have pitfalls in this respect.

For a physiological model given as a singularly perturbed system (1), there is an added complication related to a Hopf bifurcation. Suppose the full system possesses a Hopf bifurcation that persists in the singular limit as a Hopf bifurcation of the layer problem (for *k *≥ 2). We may want to know if one can relate the criticality of the Hopf bifurcation obtained in the layer problem to the criticality of the Hopf bifurcation in the full problem. Care needs to be taken because, very near the Hopf bifurcation, the time scale associated with the bifurcating directions (i.e. corresponding to the real part of the complex conjugate pair of eigenvalues) will be comparable with the time scale(s) associated with the slow variable(s), which can give rise to problems if we wish to apply GSPT.

In this article, we focus on the criticality of Hopf bifurcations in typical physiological models with multiple time scales. We show that in some cases in which a Hopf bifurcation involves the fast variables, all the information needed to determine the criticality of the bifurcation is contained in the fast subsystem but in other cases there is crucial information in the slow dynamics that can change the criticality of the Hopf bifurcation, a seemingly counterintuitive result.

The outline of this article is as follows. In section 2, we look at a model reduction technique widely used in the analysis of physiological models that can be written as slow-fast systems, and determine conditions under which the use of this technique can be rigorously justified by centre manifold theory. In section 3, we focus on Hopf bifurcations in slow-fast systems. After reviewing the general procedure for computing the criticality of a Hopf bifurcation (Section 3.1), we show that the physiological model reduction technique considered in Section 2 can change the criticality of a Hopf bifurcation, so that the criticality of a Hopf bifurcation in a model may not match the criticality of the corresponding Hopf bifurcation in the reduced model (Section 3.2). We go on to show that there are potential traps in determining the criticality of a Hopf bifurcation when we try to apply GSPT; the criticality of a Hopf bifurcation in the layer problem may not match that of the corresponding Hopf bifurcation in the full system (Section 3.3), although matters are more straightforward when there is no Hopf bifurcation in the layer problem (Sections 3.4 and 3.5). We illustrate our results with numerical examples throughout Section 3. Section 4 contains some conclusions.

## 2 A physiological model reduction technique for slow-fast systems

In this section, we outline a model reduction technique widely used in physiological models that are modelled as slow-fast systems, and find conditions under which the use of this technique is justified. Many physiological models, including many neural and calcium models, contain gating variables *m *= (*m*_1_,..., *m_j_*) which are thought to evolve on a time scale which is fast compared with other processes. In these cases, a classic first step is to set the fast gating variables to their quasi-steady-state values, and thereby reduce the dimension of the model by the number of gating variables treated in this way. In this section, we show that this procedure can sometimes be justified by centre and invariant manifold theory.

Specifically, we are concerned with physiological models that are described in dimensionless form by singularly perturbed systems of the form

(3) $$\begin{array}{c} v^{\prime }=f\left(v,m,n,\mu ,\varepsilon \right),\\ m^{\prime }=h\left(v,m,n,\mu ,\varepsilon \right),\\ n^{\prime }=\varepsilon g\left(v,m,n,\mu ,\varepsilon \right), \end{array}$$

where (*v*, *m*) ∈ ℝ × ℝ*^j ^*= ℝ*^k ^*are the fast variables, *n *∈ ℝ*^l ^*are the slow variables, *f*, *g *and *h *are order-one vector-valued functions, *μ *∈ ℝ*^m ^*are system parameters, prime denotes differentiation with respect to the fast time *t *and *ε *≪ 1 is the singular perturbation parameter reflecting the time scale separation. In neural models, *v *will typically represent voltage, while in calcium models, *v *might represent the cytosolic calcium concentration. In biophysical (conductance-based) models, *m *represents the fast gating variables and *n *represents the slow gating variables. In calcium models, the total calcium concentration might also be included in the slow variables *n*.

By taking the singular limit *ε *→ 0 in (3), we obtain the layer problem, which possesses, in general, an *l*-dimensional manifold of equilibria called the critical manifold,^b^

$$S_{0}:=\left\{\left(v,m,n\right):f\left(v,m,n,\mu ,0\right)=h\left(v,m,n,\mu ,0\right)=0\right\}.$$

We are interested in different cases, depending on whether or not the critical manifold is normally hyperbolic, and, if it is not normally hyperbolic, the way in which it fails to be normally hyperbolic.

**Assumption 1 ***The critical manifold S*_0 _*is normally hyperbolic, i.e. all eigenvalues of the *(*k *× *k*) *Jacobian matrix of the layer problem evaluated along S*_0_,

$$J={\left(\left(\begin{array}{cc} \frac{\partial }{\partial v}f & D_{m}f\\ \frac{\partial }{\partial v}h & D_{m}h \end{array}\right)\right|}_{S_{0}},$$

*have real parts not equal to zero*.

Fenichel theory [2,3] applies under this assumption and we have the following result:

**Proposition 1 ***Given system *(3) *under Assumption 1, then there exists an l-dimensional invariant manifold S*_*ε *_*given as a graph *$\left(v,m\right)=\left(\hat{V}\left(n,\mu ,\varepsilon \right),\hat{M}\left(n,\mu ,\varepsilon \right)\right)$. *This invariant manifold is a smooth O*(*ε*) *perturbation of S*_0_. *System *(3) *reduced to S*_*ε *_*has the form*

(4) $$ṅ=g\left(\hat{V}\left(n,\mu ,\varepsilon \right),\hat{M}\left(n,\mu ,\varepsilon \right),n,\mu ,\varepsilon \right),$$

*where the overdot denotes differentiation with respect to the slow time scale τ *= *εt. Since S_ε _is a regular perturbation of S*_0_, *the slow flow *(4) *on S_ε _is a regular O*(*ε*) *perturbation of the reduced flow on S*_0 _*given by*

(5) $$ṅ=g\left(\hat{V}\left(n,\mu ,0\right),\hat{M}\left(n,\mu ,0\right),n,\mu ,0\right).$$

If we assume that *S*_0 _is normally hyperbolic with all eigenvalues having real part less than 0, then Proposition 1 implies that a model reduction onto the slow manifold *S_ε _*will cover the dynamics of the model after some initial transient time. In a biophysical model that would imply that the reduction of the fast gating variables *m *and, e.g., voltage or cytosolic calcium concentration *v *to their quasi-steady-state values is correct to leading order of the perturbation, i.e. it correctly describes the flow on *S*_0_.

Unfortunately, most physiological models have a critical manifold that is not normally hyperbolic and the reduction technique that Proposition 1 suggests is not (globally) justified. In the following, we focus on the two main cases that cause loss of normal hyperbolicity of *S*_0_: a fold or a Hopf bifurcation in the layer problem.

**Assumption 2 ***The Jacobian of the layer problem evaluated along S*_0_, *i.e. the *(*k *× *k*)*-matrix*

$$J={\left(\left(\begin{array}{cc} \frac{\partial }{\partial v}f & D_{m}f\\ \frac{\partial }{\partial v}h & D_{m}h \end{array}\right)\right|}_{S_{0}},$$

*has a zero eigenvalue along F *:= {(*v*, *m*, *n*) ∈ *S*_0 _: det(*J*) = 0, *rank *(*J*) = *j*= *k *- 1} *which is an *(*l *- 1)*-dimensional subset of S*_0_. *We further assume that the other j eigenvalues all have real parts less than 0 along S*_0_.

Generically, the manifold *S*_0 _is folded near *F *if the following non-degeneracy conditions are fulfilled (evaluated along *F*):

(6) $$w_{l}\cdot \left[\left(D_{\left(v,m\right)\left(v,m\right)}^{2}\left(f,h\right)\right)\left(w_{r},w_{r}\right)\right]\neq 0,\;w_{l}\cdot \left[D_{n}\left(f,h\right)\right]\neq 0$$

where *w*_l _and *w*_r _denote the left and right null vectors of the Jacobian *J*. Without loss of generality, we assume that the (*j *× *j*) sub-matrix *D_m_h *of the Jacobian *J *has full rank *j*. This implies that the right nullvector *w*_r _of *J *has a non-zero *v*-component, i.e. the nullspace is not in *v *= 0.

Next we make use of the fact that the determinant of the Jacobian *J *can be calculated by

$$\det\left(J\right)=\det\left(D_{m}h\right)\cdot \det\left(\frac{\partial }{\partial v}f-D_{m}f{\left(D_{m}h\right)}^{-1}\frac{\partial }{\partial v}h\right)$$

which follows from the block structure of *J *and the Leibniz formula for determinants. By Assumption 2, det(*J*) = 0 along *F*. Since *D_m_h *has full rank, det(*D_m_h*) ≠ 0 along *F*. Hence, the second determinant

$$\det\left(\frac{\partial }{\partial v}f-D_{m}f{\left(D_{m}h\right)}^{-1}\frac{\partial }{\partial v}h\right)=0$$

along *F *which implies that $\frac{\partial }{\partial v}f-D_{m}f{\left(D_{m}h\right)}^{-1}\frac{\partial }{\partial v}h=0$ along *F *(because it is a scalar). This reflects the zero eigenvalue of *J*. Since det(*D_m_h*) ≠ 0, it also follows from the *implicit function theorem *that *h*(*v*, *m*, *n*, *μ*, *ε*) = 0 can be solved for *m *= *M*(*v*, *n*, *μ*, *ε*). Note that in neural models this functional relation is automatically given by the quasi-steady-state functions *m_i _*= *M_i_*(*v*, *n*, *μ*, *ε*) = *m_i_*,_∞_(*v*), *i *= 1,..., *j*, for the fast gating variables.

In the following, we generalise a result that was presented in [19] for the HH model (compare also with general results on systems with folded critical manifolds in [9]).

**Proposition 2 ***Given system *(3) *under Assumption 2, then there exists an *(*l*+1)*-dimensional centre manifold W^c ^in a neighbourhood of the fold F given as a graph *$m=\hat{M}\left(v,n,\mu ,\varepsilon \right)$. *System *(3) *reduced to W^c ^has the form*

(7) $$\begin{array}{c} v^{\prime }=f\left(v,\hat{M}\left(v,n,\mu ,\varepsilon \right),n,\mu ,\varepsilon \right),\\ n^{\prime }=\varepsilon g\left(v,\hat{M}\left(v,n,\mu ,\varepsilon \right),n,\mu ,\varepsilon \right). \end{array}$$

Since the right nullvector *w*_r _has a non-zero *v*-component it follows that the one-dimensional centre manifold of the layer problem of (3) is (locally) given as a graph over the *v*-space. Thus, the corresponding (*l *+ 1)-dimensional centre manifold of the full system (3) is also (locally) given as a graph $m=\hat{M}\left(v,n,\mu ,\varepsilon \right)$. Introducing the nonlinear coordinate transformation $\hat{m}=m-\hat{M}\left(v,n,\mu ,\varepsilon \right)$ to system (3) gives

(8) $$\begin{array}{ll} v^{\prime } & =f\left(v,m\left(v,\hat{m},n,\mu ,\varepsilon \right),n,\mu ,\varepsilon \right),\;\\ {\hat{m}}^{\prime } & =h\left(v,m\left(v,\hat{m},n,\mu ,\varepsilon \right),n,\mu ,\varepsilon \right)-\frac{\partial }{v}\hat{M}\left(v,n,\mu ,\varepsilon \right)f\left(v,m\left(v,\hat{m},n,\mu ,\varepsilon \right),n,\mu ,\varepsilon \right),\;\\ & \;-\varepsilon D_{n}\hat{M}\left(v,n,\mu ,\varepsilon \right)g\left(v,m\left(v,\hat{m},n,\mu ,\varepsilon \right),n,\mu ,\varepsilon \right),\;\\ n^{\prime } & =\varepsilon g\left(v,m\left(v,\hat{m},n,\mu ,\varepsilon \right),n,\mu ,\varepsilon \right),\;\\ & \; \end{array}$$

where the (*l *+ 1)-dimensional centre manifold is now aligned with $\hat{m}=0$. Hence, the flow on the (*l *+ 1)-dimensional centre manifold is given by system (7). This proves the assertion. □

Note that, in general, $M\neq \hat{M}$, i.e. solving the equation *h*(*v*, *m*, *n*, *μ*, *ε*) = 0 for *m *= *M*(*v*, *n*, *μ*, *ε*) does not yield the centre manifold for any *ε*, including *ε *= 0. Thus, the dynamics of the reduced system obtained using the quasi-steady-state reduction is, in general, different to the dynamics of the full system reduced to the centre manifold. The difference between *M *and $\hat{M}$ is due to two terms: an *ε*-dependent term that tends to zero in the singular limit and a term that is due to *f*. This last term will vanish on the critical manifold (where *f *= 0) and so on the critical manifold, $M\to \hat{M}$ as *ε *→ 0.

In summary, we have shown that making a quasi-steady-state approximation can be mathematically justified if the critical manifold is normally hyperbolic (Proposition 1) or if it loses normal hyperbolicity in a simple fold and we are concerned with dynamics near the fold only (Proposition 2). In these cases, quantitative changes may be introduced by the approximation but the qualitative features of the dynamics will be preserved.

### 2.1 The Hodgkin-Huxley model

As an example of such a model reduction, we look again at the HH model which models the space-clamped squid giant axon. This model is a four-dimensional system that in dimensionless form is given by

(9) $$\begin{array}{ll} \varepsilon v^{\prime } & =Ī-m^{3}h\left(v-{Ē}_{Na}\right)-{ḡ}_{k}n^{4}\left(v-{Ē}_{k}\right)-{ḡ}_{l}\left(v-{Ē}_{L}\right)\equiv S\left(v,m,n,h\right),\;\\ \varepsilon m^{\prime } & =\frac{1}{\tau_{m}t_{m}\left(v\right)}\left(m_{\infty }\left(v\right)-m\right)\equiv M\left(v,m\right),\;\\ h^{\prime } & =\frac{1}{\tau_{h}t_{h}\left(v\right)}\left(h_{\infty }\left(v\right)-h\right)\equiv H\left(v,h\right),\;\\ n^{\prime } & =\frac{1}{\tau_{n}t_{n}\left(v\right)}\left(n_{\infty }\left(v\right)-n\right)\equiv N\left(v,n\right),\;\\ & \; \end{array}$$

where the fast variables are *v *and *m *(dimensionless membrane potential and activation gate of the sodium channel) and the slow variables are *h *and *n *(inactivation gate of the sodium channel and activation gate of the potassium channel). The quantity  is the bifurcation parameter (and is proportional to the applied external current *I*), and expressions for the functions *m*_∞_(*v*), *n*_∞_(*v*), *h*_∞ _(*v*), etc. and the values of constants used in (9) are given in the Appendix.

It was shown in [19] that the two-dimensional critical manifold is cubic-shaped in the physiologically relevant domain of the phase space, with two fold-curves *F*^±^, attracting outer branches and a middle branch of saddle type. Furthermore, the vector field has a three-dimensional centre manifold $m=\hat{M}\left(v,n,h,\varepsilon \right)$ along each fold curve *F*^±^, which is exponentially attracting. Hence, Proposition 2 can be applied and the vector field reduced to the centre manifold near each fold *F*^± ^is given by

(10) $$\begin{array}{ll} \varepsilon v^{\prime } & =Ī-{\hat{M}}^{3}\left(v,n,h,\varepsilon \right)h\left(v-{Ē}_{Na}\right)-{ḡ}_{k}n^{4}\left(v-{Ē}_{k}\right)-{ḡ}_{l}\left(v-{Ē}_{L}\right),\;\\ h^{\prime } & =\frac{1}{\tau_{h}t_{h}\left(v\right)}\left(h_{\infty }\left(v\right)-h\right),\;\\ n^{\prime } & =\frac{1}{\tau_{n}t_{n}\left(v\right)}\left(n_{\infty }\left(v\right)-n\right).\;\\ & \; \end{array}$$

One of the classical reduction steps in the literature is to use the quasi-steady-state approximation *m *= *m*_∞_(*v*) rather than perform the full centre manifold reduction $m=\hat{M}$ shown above. We have to expect quantitative changes in the reduced model (i.e. in Equations (10) with $\hat{M}\left(v,n,h,\varepsilon \right)$ replaced by *m*_∞_(*v*)) compared to the full HH model (9), and such changes are in fact observed. For example, (9) has a subcritical Hopf bifurcation for *I *= 9.8 *μ*A/cm^2 ^(i.e. Ī=0.00082) while (10) with $\hat{M}=m_{\infty }$ has a subcritical Hopf bifurcation for *I *= 7.8 *μ*A/cm^2 ^(i.e. Ī=0.00065). We note that the Hopf bifurcation of (9) is in the vicinity of the fold curve for sufficiently small *ε*, because in the singular limit the bifurcation is a *singular Hopf bifurcation *[19,24]. Thus, the Hopf bifurcation in (9) is in the regime covered by Proposition 2. Further discussion of this type of Hopf bifurcation is contained in Section 3.4.

## 3 Hopf bifurcation in slow-fast systems

In the previous section, it was shown that the quasi-steady-state reduction technique is mathematically justified in a slow-fast system if the critical manifold is normally hyperbolic or if we are interested in the dynamics near a simple fold of the critical manifold. In this section, we show that the model reduction technique discussed above, when applied to slow-fast systems with a Hopf bifurcation, may lead to changes in the criticality of the Hopf bifurcation. From a dynamical systems point of view, it is well established that misleading results can be obtained if a proper centre manifold reduction is not performed prior to the identification of bifurcations [22,23]. However, in the context of biophysical systems, model variables often have a direct physiological meaning and so it is tempting to try to avoid making coordinate transformations that combine the variables into physically ambiguous combinations. (Transformations required for centre manifold reductions are frequently of this type.) Unfortunately, this has resulted in some erroneous conclusions in the literature about the criticality of Hopf bifurcations in some biophysical models, as we will show in this section.

We then go on to show that there can be problems with the use of GSPT in analysing models with Hopf bifurcations, and in particular show that the criticality of a Hopf bifurcation in a full slow-fast system may not match the criticality of the corresponding Hopf bifurcation in the associated layer problem. This last result is independent of whether a quasi-steady-state assumption or other reduction technique has been used prior to applying GSPT.

### 3.1 Computing the criticality of a Hopf bifurcation

We first give a brief review of the general procedure for computing the criticality of a Hopf bifurcation. The criticality of a Hopf bifurcation is determined by the sign of the *first Lyapunov coefficient *of a system near a Hopf bifurcation [23,25,26]. Specifically, consider a general system

$$x^{\prime }=f\left(x;\mu \right),$$

with *x *∈ ℝ*^n^*, *μ *∈ ℝ and with a Hopf bifurcation at *x *= 0, $\mu =\hat{\mu }$. Write the Taylor expansion of $f\left(x;\hat{\mu }\right)$ at *x *= 0 as

$$f\left(x;\hat{\mu }\right)=Ax+\frac{1}{2}B\left(x,x\right)+\frac{1}{6}C\left(x,x,x\right)+O\left(||x|{|}^{4}\right),$$

where *A *is the Jacobian matrix evaluated at the bifurcation, and *B*(*x*, *y*) and *C*(*x*, *y*, *z*) are multilinear functions with components

(11) $$B_{j}\left(x,y\right)=\overset{n}{\underset{k,l=1}{\sum }}{\left(\frac{\partial^{2}f_{j}\left(\xi ;\hat{\mu }\right)}{\partial \xi_{k}\partial \xi_{l}}\right|}_{\xi =0}x_{k}y_{l},$$

(12) $$C_{j}\left(x,y,z\right)=\overset{n}{\underset{k,l,m=1}{\sum }}{\left(\frac{\partial^{3}f_{j}\left(\xi ;\hat{\mu }\right)}{\partial \xi_{k}\partial \xi_{l}\partial \xi_{m}}\right|}_{\xi =0}x_{k}y_{l}z_{m},$$

where *j *= 1, 2,..., *n*. Let *q *∈ ≤*^n ^*be a complex eigenvector of *A *corresponding to the eigenvalue *iω*, i.e. *Aq *= *iωq*. Let *p *be the associated adjoint eigenvector, i.e. *p *∈ ≤*^n ^*and *A^T^p *= *-iωp*, 〈*p*, *q*〉 = 1. Here $\langle p,q\rangle ={\bar{p}}^{T}q$ is the usual inner product in ≤*^n^*. Then the first Lyapunov coefficient for the system is defined as

(13) $$\begin{array}{ll} l_{1}=\frac{1}{2\omega }Re & \left[\langle p,C\left(q,q,\bar{q}\right)\rangle -2\langle p,B\left(q,A^{-1}B\left(q,\bar{q}\right)\right)\rangle \right)\;\\ & \left(+\langle p,B\left(\bar{q},{\left(2i\omega I_{n}-A\right)}^{-1}B\left(q,q\right)\right)\rangle \right],\;\\ & \; \end{array}$$

where *I_n _*is the *n *× *n *identity matrix. If *l*_1 _*<*0 the Hopf bifurcation is supercritical and produces periodic solutions that are stable on the two-dimensional centre manifold corresponding to the Hopf bifurcation. If *l*_1 _*>*0, the Hopf bifurcation is subcritical and the associated periodic orbits are unstable within the centre manifold.

### 3.2 Hopf bifurcations and model reduction

Here we are concerned with physiological models that are of the same form as (3) except that *v *is now in ℝ^2 ^instead of in ℝ. Specifically, we are interested in models that are described in dimensionless form by singularly perturbed systems of the form

(14) $$\begin{array}{c} v^{\prime }=f\left(v,m,n,\mu ,\varepsilon \right),\\ m^{\prime }=h\left(v,m,n,\mu ,\varepsilon \right),\\ n^{\prime }=\varepsilon g\left(v,m,n,\mu ,\varepsilon \right), \end{array}$$

where (*v*, *m*) ∈ ℝ^2^×ℝ*^j ^*= ℝ*^k ^*are the fast variables, *n *∈ ℝ*^l ^*are the slow variables, *f*, *g *and *h *are order-one vector-valued functions, *μ *∈ ℝ*^m ^*are system parameters and *ε *≪ 1 is the singular perturbation parameter reflecting the time scale separation. Without loss of generality, we fix *m *- 1 parameters, and consider Hopf bifurcations that occur as the other parameter, which we denote by *ν*, is varied.

**Assumption 3 ***System *(14) *possesses a non-degenerate Hopf bifurcation at *$\nu ={\hat{\nu }}_{\varepsilon }$. *Specifically, for sufficiently small ε:*

*(a) there exists a family of equilibria*(*v*(*ν*,*ε*), *m*(*ν*,*ε*), *n*(*ν*,*ε*)), *for ν in a neighbourhood of *${\hat{\nu }}_{\varepsilon }$, *such that the Jacobian matrix has a pair of eigenvalues, λ*_1_(*ν*) *and λ*_2_(*ν*), *with *$\lambda_{1}\left({\hat{\nu }}_{\varepsilon }\right)={\bar{\lambda }}_{2}\left({\hat{\nu }}_{\varepsilon }\right)=i\omega$*where ω *= *O*(1), *while the other *(*k *- 2) *eigenvalues associated with the fast components of the vector field all have real parts of order O*(1), *which we assume to be negative*,

*(b) *$\frac{d}{d\nu }Re\left(\lambda_{1}\right){|}_{\nu ={\hat{\nu }}_{\varepsilon }}=O\left(1\right)\neq 0$;

*(c) l*_1_(*ε*) = *O*(1) ≠ 0, *where l*_1 _*is the first Lyapunov coefficient associated with the Hopf bifurcation;*

*(d) the bifurcation parameter ν persists in the singular limit ε *→ 0, *i.e. ν appears explicitly in the layer problem*.

The condition *ω *= *O*(1) ensures that the Hopf bifurcation is in the fast variables. Thus, there is a Hopf bifurcation for $\nu ={\hat{\nu }}_{0}$ in the singular limit system of (14), the layer problem.^c ^We assume, without loss of generality, that the complex eigenvector *q *∈ ≤*^k ^*of the eigenvalue *iω *in the layer problem of (14) has non-zero entries in the first two fast components of the vector field, *v *∈ ℝ^2^, i.e. we associate the Hopf bifurcation with the direction of *v*.

A natural first step in determining the criticality of the Hopf bifurcation in the full system (14) might be to reduce the dimension of the model by setting the fast gating variables *m *∈ ℝ*^j ^*to their quasi-steady state as described in Section 2. Since *D_m_h *is invertible we can invoke the implicit function theorem and solve *h *= 0 for *m *= *M*(*v*, *n*, *μ*, *ε*). Again, we can introduce a coordinate change $\hat{m}=m-M\left(v,n,\mu ,\varepsilon \right)$. However, this process need not correspond to a proper centre manifold reduction as in the case of a folded critical manifold. In general, one also has to introduce new coordinates $\hat{v}\in {\mathbb{R}}^{2}$ to align the centre manifold with $\hat{m}=0$. Hence, a reduction of the fast gating variables *m *alone typically changes the first Lyapunov coefficient which might change the criticality of the Hopf bifurcation, so that the Hopf bifurcation in the full system is subcritical while the Hopf bifurcation in the lower-dimensional system is supercritical (or vice versa). This effect is independent of whether the Hopf bifurcation involves fast or slow variables.

#### 3.2.1 The Chay-Keizer model

**Figure 1:**
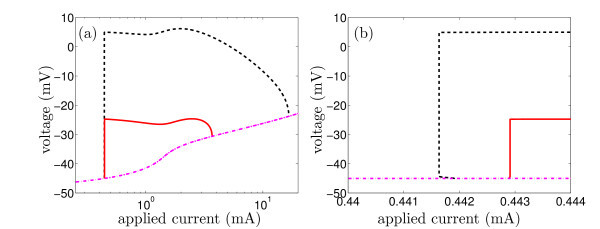
**Bifurcation diagrams for two versions of the Chay-Keizer model: the full five-dimensional model, Equations (15) and the reduced three-dimensional model obtained by setting *m *and *h *equal to their quasi-steady-state values**. The position of the equilibrium solutions is the same in both models and is indicated by the pink dot-dash curve. The black dashed curve shows the maximum voltage attained on a branch of periodic solutions in the full model, while the red solid curve shows the maximum voltage attained on the corresponding branch of periodic orbits in the reduced model. **(b) **An enlargement of part of **(a)**, near the left pair of Hopf bifurcations.

An example in which we get such a change of criticality is the Chay-Keizer model of a pancreatic *β*-cell [27]. This minimal biophysical model was originally developed as a system of five ordinary differential equations:

(15) $$\begin{array}{ll} C_{m}\frac{dV}{dt} & =-I_{Ca}\left(V\right)-\left({ḡ}_{K}n^{4}+\frac{{ḡ}_{K,Ca}c}{K_{d}+c}\right)\left(V-V_{K}\right)-{ḡ}_{L}\left(V-V_{L}\right)+I_{app},\;\\ \frac{dn}{dt} & =a_{n}\left(1-n\right)-b_{n}n,\;\\ \frac{dm}{dt} & =a_{m}\left(1-m\right)-b_{m}m,\;\\ \frac{dh}{dt} & =a_{h}\left(1-h\right)-b_{h}h,\;\\ \frac{dc}{dt} & =f\left(-k_{1}I_{Ca}\left(V\right)-k_{c}c\right),\;\\ & \; \end{array}$$

where *V *represents the membrane potential, *n *the activation gate of a potassium channel, *m *and *h *the activation and inactivation gates of a calcium channel and *c *the cytosolic concentration of free calcium. The quantity $I_{Ca}\left(V\right)={ḡ}_{Ca}m^{3}h\left(V-V_{Ca}\right)$ is the calcium current and *I*_app _is an applied external current and is also the bifurcation parameter. The other parameter values and the functions *a_n_*, *b_n_*, etc. are specified in the Appendix. A straightforward numerical bifurcation analysis of system (15) using the software package AUTO [28] shows that there are two Hopf bifurcations, with a subcritical Hopf bifurcation at *I*_app _≈ 0.4419, as shown in Figure 1.

On the other hand, in [29], the authors simplify the five-dimensional Chay-Keizer model by setting the gating variables *m *and *h *equal to their quasi-steady-state values, i.e. they choose

$$\begin{array}{c} m=\frac{a_{m}\left(V+V^{\prime }\right)}{a_{m}\left(V+V^{\prime }\right)+b_{m}\left(V+V^{\prime }\right)}:=m_{\infty }\left(V\right),\\ h=\frac{a_{h}\left(V+V^{\prime }\right)}{a_{h}\left(V+V^{\prime }\right)+b_{h}\left(V+V^{\prime }\right)}:=h_{\infty }\left(V\right) \end{array}$$

assuming implicitly that these gates have fast kinetics. Numerical bifurcation analysis of the corresponding three-dimensional system that results from this process reveals that this reduced model has a supercritical Hopf bifurcation at *I*_app _≈ 0.4429. Thus, the reduction of the dimension of this system by removing (fast) gating variables changes the criticality of the Hopf bifurcation^d^; if an aim of analysis is to determine the criticality of Hopf bifurcations, then this type of reduction should not be attempted.^e^

Figure 1 also shows that both versions of the model have a second Hopf bifurcation at much higher applied current. In both cases, this is a supercritical bifurcation but the value of the parameter at the bifurcation differs significantly between the models. Thus, the model reduction used also has the effect of making a significant change to the amplitude of the oscillations and the range of values of the applied current for which the oscillations occur.

### 3.3 Hopf bifurcation in the full slow-fast system versus the layer problem

A second potential trap in determining the criticality of a Hopf bifurcation in system (14) comes when we try to apply GSPT. From Assumption 3 it follows that a Hopf bifurcation in the full system will persist in the singular limit as a Hopf bifurcation in the layer problem. It might be tempting to proceed by determining the nature of the Hopf bifurcation in the layer problem and then asserting that the Hopf bifurcation in the slow-fast system will be of the same type. However, the existence of a Hopf bifurcation satisfying Assumption 3 automatically implies that the critical manifold of the full system is not normally hyperbolic near the bifurcation, and, hence, that Fenichel theory [2] is not applicable. In this case, there is no guarantee that complete information about bifurcations in the full system can be obtained from analysis of the layer problem alone.

Let us revisit the Chay-Keizer model (15). If we assume that *c *is a slow variable and (*v*, *m*, *h*, *n*) are fast variables, as is usually done in the literature, then the layer problem is four-dimensional. Numerical bifurcation analysis of the layer problem shows that it has a supercritical Hopf bifurcation at *I*_app _≈ 0.4427. Again, the criticality of the Hopf bifurcation has changed: the criticality of the Hopf bifurcation in the layer problem is not the same as the criticality of the corresponding Hopf bifurcation in the full system. At first glance, this result seems counterintuitive since one does not expect that the small (*O*(*ε*)) terms of the slow *c *equation in (15) play an important role in the calculation of the first Lyapunov coefficient.

In the following, we show how these small *O*(*ε*) terms can be significant in determining the criticality of a Hopf bifurcation in a slow-fast system. In particular, we show that calculating the first Lyapunov coefficient *l*_1_(*ε*) of a Hopf bifurcation in the full system and then taking the limit *ε *→ 0 does not give the Lyapunov coefficient ${\hat{l}}_{1}$ of the Hopf bifurcation in the corresponding layer problem, i.e. in general,

(16) $$\underset{\varepsilon \to 0}{\lim}l_{1}\left(\varepsilon \right)\neq {\hat{l}}_{1}.$$

#### 3.3.1 First Lyapunov coefficient for a three-dimensional problem

Consider the system of equations

(17) $$\begin{array}{c} x^{\prime }=f_{1}\left(x,y,z;\mu ,\varepsilon \right),\\ y^{\prime }=f_{2}\left(x,y,z;\mu ,\varepsilon \right),\\ z^{\prime }=\varepsilon g\left(x,y,z,\mu ,\varepsilon \right), \end{array}$$

where *x*, *y*, *z *∈ ℝ, *μ *∈ ℝ is the bifurcation parameter, *ε *is a small parameter and *f*_1_, *f*_2 _and *g *are *O*(1) smooth functions. Then, *x *and *y *are fast variables and *z *is a slow variable. Suppose that Assumption 3 is fulfilled for system (17) -- thus system (17) and the corresponding layer problem both have Hopf bifurcations. Furthermore, we assume that $\left(0,0;0,{\hat{\mu }}_{0},0\right)$ is the Hopf point of the layer problem. Note that the position of the Hopf point in phase and parameter space can vary with *ε*, by *O*(*ε*), and so the Hopf bifurcation value $\mu ={\hat{\mu }}_{\varepsilon }$ of the full system is, in general, different to the bifurcation value $\mu ={\hat{\mu }}_{0}$ of the layer problem. More importantly, we show that the *O*(*ε*) terms in the slow equation can produce an *O*(1) change in the first Lyapunov coefficient which in turn may lead to a change of the criticality of the Hopf bifurcation in the full system compared with the criticality of the associated Hopf bifurcation in the layer problem. This means that analysis of the layer problem alone is not sufficient to determine the dynamics associated with the Hopf bifurcation.

Since the Hopf point of the layer problem is $\left(0,0;0,{\hat{\mu }}_{0},0\right)$, it is straightforward to use the formulae in Section 3.1 to compute the first Lyapunov coefficient (13) for the Hopf bifurcation in the layer problem for (17), i.e. for the system

(18) $$\begin{array}{c} x^{\prime }=f_{1}\left(x,y;z,\mu ,0\right),\\ y^{\prime }=f_{2}\left(x,y;z,\mu ,0\right). \end{array}$$

It is convenient for what follows to introduce some notation. The Jacobian matrix, *A_a_*, at the Hopf point, and its inverse $A_{a}^{-1}$ are

$$A_{a}=\left(\begin{array}{cc} a_{11} & a_{12}\\ a_{21} & a_{22} \end{array}\right),\;A_{a}^{-1}=\frac{1}{\omega_{a}^{2}}\left(\begin{array}{cc} a_{22} & -a_{12}\\ -a_{21} & a_{11} \end{array}\right),$$

with *a*_11 _+ *a*_22 _= 0 and $a_{11}a_{22}-a_{21}a_{12}\equiv \omega_{a}^{2}>0$. Let *q_a _*= (*q*_1_, *q*_2_) be a (right) eigenvector of *A_a _*corresponding to the eigenvalue *iω_a _*and let *p_a _*= (*p*_1_, *p*_2_) be the corresponding adjoint (or left) eigenvector. Then, defining *B_a _*and *C_a _*as in Section 3.1 (with the subscript merely denoting that they are the *B *and *C *multilinear forms corresponding to the same system as *A_a_*), the first Lyapunov coefficient (13) is

$$\begin{array}{c} {\hat{l}}_{1a}=\frac{1}{2\omega_{a}}Re\left[\langle p_{a},C_{a}\left(q_{a},q_{a},\bar{q_{a}}\right)\rangle -2\langle p_{a},B_{a}\left(q_{a},A_{a}^{-1}B_{a}\left(q_{a},\bar{q_{a}}\right)\right)\rangle \right)\\ \;+\left(\langle p_{a},B_{a}\left(\bar{q_{a}},{\left(2i\omega_{a}I_{2}-A_{a}\right)}^{-1}B_{a}\left(q_{a},q_{a}\right)\right)\rangle \right]. \end{array}$$

We now return to the full system (17). The Jacobian matrix at the Hopf point will have the form

$$A_{c}=\left(\begin{array}{ccc} a_{11}+O\left(\varepsilon \right) & a_{12}+O\left(\varepsilon \right) & a_{13}\\ a_{21}+O\left(\varepsilon \right) & a_{22}+O\left(\varepsilon \right) & a_{23}\\ \varepsilon a_{31} & \varepsilon a_{32} & \varepsilon a_{33} \end{array}\right),$$

where the matrix has a purely imaginary eigenvalue *iω_c _*with $\omega_{c}^{2}=\omega_{a}^{2}+O\left(\varepsilon \right)$. The inverse matrix $A_{c}^{-1}$ is given by

$$A_{c}^{-1}=\frac{O\left(1\right)}{\omega_{c}^{2}}\left(\begin{array}{ccc} a_{22}+O\left(1\right) & -a_{12}+O\left(1\right) & O\left(\varepsilon^{-1}\right)\\ -a_{21}+O\left(1\right) & a_{11}+O\left(1\right) & O\left(\varepsilon^{-1}\right)\\ \left(a_{31}+k_{1}a_{32}\right)O\left(1\right)\;\; & \left(a_{32}+k_{2}a_{31}\right)O\left(1\right)\;\; & O\left(\varepsilon^{-1}\right) \end{array}\right),$$

where *k*_1 _and *k*_2 _are *O*(1) coefficients. We note that the position of the Hopf bifurcation point can vary with *ε *and thus the entries in the corresponding (2×2)-submatrix of the Jacobian *A_c _*may differ (by at most *O*(*ε*)) from their values in the Jacobian *A_a _*of the layer problem.

A (right) eigenvector of *A_c _*corresponding to the eigenvalue *iω_c _*is given by *q_c _*= (*q*_1 _+ *O*(*ε*), *q*_2 _+ *O*(*ε*), *O*(*ε*)) with adjoint (or left) eigenvector *p_c _*= (*p*1 + *O*(*ε*), *p*_2 _+ *O*(*ε*), *p*_3 _+ *O*(*ε*)). Note that *p*_3 _= *O*(1), since it satisfies *a*_13*p*1 _+ *a*_23*p*2 _+ *iω_c_p*_3 _= 0 to leading order.

Our aim is to calculate the difference of the first Lyapunov coefficients for the full and layer problems, i.e. $l_{1c}\left(\varepsilon \right)-{\hat{l}}_{1a}$ we have $C_{c}\left(q_{c},q_{c},\bar{q_{c}}\right)=\left(O\left(1\right),O\left(1\right),O\left(\varepsilon \right)\right)$ which gives

(19) $$\langle p_{a},C_{a}\left(q_{a},q_{a},\bar{q_{a}}\right)\rangle -\langle p_{c},C_{c}\left(q_{c},q_{c},\bar{q_{c}}\right)\rangle =O\left(\varepsilon \right).$$

We also have $B_{c}\left(q_{c},\bar{q_{c}}\right)=\left(O\left(1\right),O\left(1\right),O\left(\varepsilon \right)\right)$ and $A_{c}^{-1}B_{c}\left(q_{c},\bar{q_{c}}\right)=\left(O\left(1\right),O\left(1\right),O\left(1\right)\right)$ from which follows that

(20) $$\langle p_{a},B_{a}\left(q_{a},A_{a}^{-1}B_{a}\left(q_{a},{\bar{q}}_{a}\right)\right)\rangle -\langle p_{c},B_{c}\left(q_{c},A_{c}^{-1}B_{c}\left(q_{c},{\bar{q}}_{c}\right)\right)\rangle =O\left(1\right).$$

Similarly, we obtain that

(21) $$\begin{array}{c} \langle p_{a},B_{a}\left(\bar{q_{a}},{\left(2i\omega_{a}I_{2}-A_{a}\right)}^{-1}B_{a}\left(q_{a},q_{a}\right)\right)\rangle \\ \left[0.2cm\right]-\langle p_{c},B_{c}\left(\bar{q_{c}},{\left(2i\omega_{c}I_{3}-A_{c}\right)}^{-1}B_{c}\left(q_{c},q_{c}\right)\right)\rangle =O\left(1\right). \end{array}$$

Combining all these results, we find that

(22) $$l_{1c}\left(\varepsilon \right)-{\hat{l}}_{1a}=O\left(1\right).$$

Thus, *l*_1*c *_may not tend to ${\hat{l}}_{1a}$*a *as *ε *→ 0. In other words, an *O*(*ε*) perturbation to Equations (17) can yield an *O*(1) difference in the first Lyapunov coefficient, which may induce a sign change.

It is worth having a closer look to see what causes this *O*(1) difference in (22). Note that (19) only contributes an *O*(*ε*) perturbation to the Lyapunov coefficient. Thus, third-order terms in (*x*, *y*, *z*) of the function *g *have no influence on the result. On the other hand, linear and second-order terms in (*x*, *y*, *z*) of the function *g *are responsible for the *O*(1) difference in (20) and (21). To be more precise, the quantities $\frac{\partial g}{\partial x}=a_{31}$, $\frac{\partial g}{\partial y}=a_{32}$, $\frac{\partial^{2}g}{\partial x^{2}}$, $\frac{\partial^{2}g}{\partial x\partial y}$ and $\frac{\partial^{2}g}{\partial y^{2}}$ evaluated at the Hopf bifurcation are responsible for this discrepancy. So, if these five terms do not exist, or vanish at the Hopf bifurcation, then the terms (20) and (21) are of *O*(*ε*) and the Lyapunov coefficient *l*_1*c*_(*ε*) is an *O*(*ε*) perturbation of ${\hat{l}}_{1a}$.

These results have significant consequences for computation of the criticality of the Hopf bifurcation for (17). Specifically, the *O*(1) difference found above may result in a sign change of the first Lyapunov coefficient, so that the Hopf bifurcation in the layer problem may be supercritical while the Hopf bifurcation in the full system is subcritical (or vice versa). Thus, we see that, in general, it is not possible to predict the criticality of a Hopf bifurcation in a slow-fast system with two or more fast variables in the limit *ε *→ 0 simply by observing the criticality of the associated Hopf bifurcation in the layer problem. However, in the special case that the component of the vector field associated with the slow variable is sufficiently aligned with the centre manifold of the full system (17) then there is no such difficulty; the criticality of the Hopf bifurcations in the *ε *= 0 limit of the full system and in the layer problem will match.

#### 3.3.2 Application to a model of intracellular calcium dynamics

To see how these results apply to a specific model with two fast and one slow variables, we consider a simplified version of a model of calcium oscillations [30]. In this model, oscillations in the concentration of free cytoplasmic calcium arise via sequential release and uptake of calcium to and from the endoplasmic reticulum (ER). Release of calcium from the ER is through inositol trisphosphate receptors (IPR, which are also calcium channels) and uptake of calcium into the ER is via calcium ATPase pumps, or SERCA pumps. Calcium can also enter from the outside, and is pumped out across the plasma membrane of the cell by other ATPase pumps.

In the original model of Atri et al. [30], the SERCA and plasma membrane pumps were modelled as saturating Hill functions of the calcium concentration. In addition, release of calcium through the IPR was modelled by assuming fast activation of the IPR by calcium followed by slower inactivation.

However, to construct the simplified model used here much of this complexity has been discarded, while keeping the essential qualitative features of the model. Thus, firstly, calcium release through the IPR is modelled by a combination of Hill functions, one of them delayed via the dynamic variable, *n*. The steady-state flux through the IPR is thus a biphasic function of the calcium concentration, as in the original Atri model, but the functional form is as simple as possible. Secondly, the calcium pumps are modelled as linear functions of the calcium concentration.

These assumptions result in the following model:

(23) $$\begin{array}{ll} ċ & =\left(\alpha +k_{f}\frac{c^{2}}{c^{2}+\varphi_{1}^{2}}n\right)\left(c_{t}-\left(\gamma +1\right)c\right)-k_{s}c+\varepsilon \left(J_{in}-k_{p}c\right),\;\\ ṅ & =\frac{1}{\tau }\left(\frac{\varphi_{2}}{\varphi_{2}+c}-n\right),\;\\ {ċ}_{t} & =\varepsilon \left(J_{in}-k_{p}c\right),\;\\ & \; \end{array}$$

where values of all the system parameters are given in Table 1. In this model, *c *represents the concentration of free calcium in the cytosol, *c*_t _is the total number of moles of calcium in the cell, divided by the cytoplasmic volume, and *n *is the proportion of IP_3 _receptors that have not been inactivated by calcium. This simplified version of the Atri model captures the qualitative features that are important to our discussion, but has a much simpler functional form than the full model, making it easier to work with. In this way, it bears the same relationship to a more complex calcium oscillation model as does the FitzHugh-Nagumo model to the HH model.

**Table 1:** Parameters of the simplified Atri model, Equations (23)

α	*k_s_*	*k_f_*	*k_p_*	*φ*_1_	*φ*_2_	*τ*	*γ*
0.05 s^-1^	20.0 s^-1^	20.0 s^-1^	20.0 s^-1^	2.0 *μ*M	1.0 *μ*M	2.0 s^-1^	5.0

**Figure 2:**
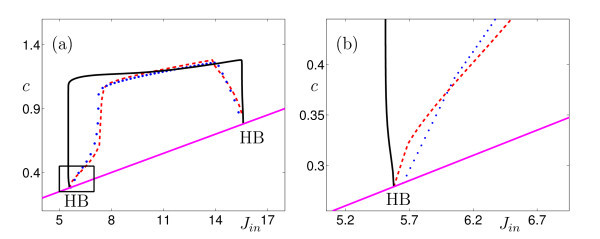
**Partial bifurcation diagram for the simplified Atri model, Equations (23) with various values of *ε *and other parameter values as in Table 1**. The pink (solid) curve shows the position of the unique equilibrium of the model. This equilibrium has two Hopf bifurcations (labelled HB), with the equilibrium being of saddle type for parameter values between the two Hopf bifurcations and being stable otherwise. The remaining curves show the maximum *c*-values attained by the periodic orbits created in the Hopf bifurcations, for three choices of *ε*, i.e. *ε *= 0 (layer problem), *ε *= 10^-4 ^and *ε *= 10^-2 ^on the black solid, red dashed and blue dotted curves, resp. **(b) **Enlargement of the marked rectangle in **(a)**. Note that the left-most Hopf bifurcation in **(a) **is subcritical when *ε *= 0 but supercritical for all *ε >*0.

The Atri model is known to be a multiple time scale system, and some results have been established about the utility of GSPT for explaining the dynamics of both the full and simplified versions of the model [31-33]. For the values of the parameters used here, the right-hand sides of the  ,  and  equations, respectively, are *O*(1), *O*(1) and *O*(*ε*), respectively, and so the model has two fast variables and one slow variable when *ε *≪ 1. (Note that *J*_in _≈ 5 at the Hopf bifurcation of interest; see Figure 2.)

Part of the bifurcation diagram for this model is shown in Figure 2, for three different choices of *ε*. The model has a unique equilibrium when *ε *≠ 0, the position of which does not depend on *ε*. This equilibrium has two Hopf bifurcations at parameter values that depend on *ε*; we are interested in the criticality of the leftmost Hopf bifurcation.

As can be seen in Figure 2, the left-most Hopf bifurcation for this model is subcritical in the layer problem but supercritical for the full problem for the two non-zero choices of *ε *shown. It can be shown that the Hopf bifurcation is in fact supercritical for all choices of small *ε*, not just the values shown. Inspection of Equations (23) shows that the differential equation for the slow variable *c*_t _contains a term that is linear in *c*, one of the fast variables, and this feature is not changed by the transformation required to shift the branch of equilibria to the origin (which is linear for the *c *component). As discussed at the end of Section 3.3.1, we can thus expect an *O*(1) difference between the first Lyapunov coefficients for the Hopf bifurcations in the layer system and the full system, and there is no reason to expect the criticality of the Hopf bifurcations to be the same for the full system and the layer problem. Knowledge of the dynamics in the layer problem is therefore insufficient to predict the criticality of the Hopf bifurcation in the full system. The Chay-Keizer model discussed above provides another example of a specific model with the same difficulty.

### 3.4 Hopf bifurcation involving both fast and slow variables

In a range of biophysical systems, including HH-type neuronal models such as system (9) and a variety of calcium models such as those in [32], Hopf bifurcations in the full system are found in the neighbourhood of a fold of the critical manifold *S*_0_, as defined by Assumption 2. In such cases, Assumption 3 is automatically violated, and neither the fast nor slow subsystem has a Hopf bifurcation; instead the Hopf bifurcation involves both fast and slow variables. This implies that the pair of complex conjugate eigenvalues associated with the Hopf bifurcation is *λ*_1 _and *λ*_2 _with $\lambda_{1}\left(\hat{\mu },\varepsilon \right)={\bar{\lambda }}_{2}\left(\hat{\mu },\varepsilon \right)=i\omega$, where $\omega =O\left(\sqrt{\varepsilon }\right)$ and so the Hopf bifurcation vanishes in the singular limit. This special type of Hopf bifurcation is known as a *singular Hopf bifurcation *[34-36] and it is closely related to the notion of canard explosion and type II folded saddle-node singularities in GSPT; we refer the reader to the literature on this subject [5,19,37]. Since the singular Hopf bifurcation vanishes in the singular limit, it is mandatory to calculate the criticality of the Hopf bifurcation for *ε *≠ 0 and we do not run into the same problem as in the previous case study; we are not tempted to use the first Lyapunov coefficient from the singular limit to predict the value of *l*_1 _in the full system, since it is zero in the singular limit and clearly non-zero in the full system.^f^

### 3.5 Hopf bifurcation in the slow subsystem

The case of Hopf bifurcation in the slow subsystem is trouble free for a singularly perturbed system (2) under Assumption 1 that the critical manifold *S*_0 _is normally hyperbolic. In this case, the eigenvalues are *λ *= ±*iω *with *ω *= *O*(*ε*), Proposition 1 applies and the slow flow on the slow manifold *S_ε _*is given by (4) which is a regular perturbation of the reduced flow (5), a remarkable insight from Fenichel's study. It now follows from the regular perturbation structure of the slow flow (4) that if we have a Hopf bifurcation in the reduced problem (5) then it persists generically as a Hopf bifurcation in the full problem (2). Furthermore, the first Lyapunov coefficient *l*_1_(*ε*) is a regular perturbation of the singular limit value *l*_1_(0). Criticality of the Hopf bifurcation in the full system is then as in the reduced problem, the slow subsystem.^g^

If, on the other hand, the critical manifold loses normal hyperbolicity at the Hopf bifurcation, then we are dealing with a more degenerate bifurcation: a 'fold-Hopf'-type bifurcation in the case where the critical manifold is folded and a 'Hopf-Hopf'-type bifurcation in the case of a simultaneous Hopf bifurcation in the layer problem. These cases are outside of the scope of this article and we do not consider them further.

## 4 Conclusions

In this article, we have discussed some difficulties that may arise when computing the criticality of Hopf bifurcations in slow-fast systems. We have identified two potential problems. The first problem may occur in neuronal-type models that include fast gating variables. In systems of this type, a typical first step in the analysis is to reduce the dimension of the model by making a quasi-steady-state assumption and replacing the differential equations for one or more of the fast gating variables by algebraic equations. This technique is widely used in the analysis of biophysical models, and in such cases is believed to preserve many important qualitative features of the dynamics. However, we have shown that this reduction technique can alter the criticality of Hopf bifurcations in the system, so that a subcritical Hopf bifurcation in the full system becomes a supercritical Hopf bifurcation in the reduced system, or vice versa. If the purpose of analysis is to determine the nature of the onset of oscillations, it may not be advisable to perform a quasi-steady-state reduction.

We note that a change in the criticality of the Hopf bifurcation alone may not make a large change to the overall observed dynamics. For instance, in the Chay-Keizer model discussed in Section 3.2.1, the branch of periodic solutions near the left-most Hopf bifurcation is very steep, in both the full system and the reduced system obtained by applying a quasi-steady-state assumption (see Figure 1). This means that the onset of stable oscillations occurs at almost the same parameter value in both versions of the model, despite the criticalities of the Hopf bifurcations being different. Note, however, that in this model the amplitude of the oscillations is very different in the two models, as is the overall parameter range for which oscillations exist.

The second potential problem we discussed may arise if we attempt to use GSPT in the analysis of a model with a Hopf bifurcation. GSPT aims to use lower-dimensional fast and slow subsystems to make predictions about the dynamics in the full system. We have shown that when a Hopf bifurcation in a (full) slow-fast system has a corresponding Hopf bifurcation in the layer problem (i.e. the equilibrium has eigenvalues *λ *= ±*iω *with *ω *= *O*(1)) the criticality can differ between the full system and the fast subsystem. This means that the layer problem cannot be used to make predictions about the criticality of the Hopf bifurcation in the full system. In some biophysical models, the layer problem corresponds to a physically distinct state of the system. For example, in models of intracellular calcium dynamics, the layer problem frequently can be thought of a modelling the cell with no flux across the cell membrane. In such a situation, it is tempting to presume that the dynamics of the layer problem will match the dynamics of the full model in the limit that we approach the layer problem. We have shown that this is not the case, at least for the criticality of Hopf bifurcations.

There are no such difficulties in computing the criticality of Hopf bifurcations that involve slow variables. We discussed two cases. The first case occurs when the Hopf bifurcation in the full model is caused by the interaction of a slow and a fast variable. In this case, the Hopf bifurcation is a singular Hopf, in which case the Hopf bifurcation vanishes in the singular limit (i.e. the relevant eigenvalues for the Hopf bifurcation are *λ *= ±*iω *with $\omega =O\left(\sqrt{\varepsilon }\right)$, and one is not tempted to deduce the criticality of the Hopf bifurcation in the full problem from the dynamics of the layer problem (or the reduced problem). Alternatively, if the critical manifold is normally hyperbolic and there is a Hopf bifurcation in the reduced problem (i.e. *λ *= ±*iω *with *ω *= *O*(*ε*)), the criticality of the Hopf bifurcation will be the same in the full system and the reduced problem.

In recent study [38], Guckenheimer and Osinga investigate two slow-fast systems in which the criticality of a Hopf bifurcation in the full system does not match the criticality of the corresponding Hopf bifurcation in the layer problem. They show that in each case there is a nearby torus bifurcation in the slow-fast system, and that the family of periodic orbits in the full system is *O*(*ε*) close to the family of periodic orbits in the layer problem, regardless of the criticality of the Hopf bifurcation. A practical consequence of their study is that observation of a torus bifurcation close to a Hopf bifurcation in a slow-fast system is a possible indication that the full system and the corresponding layer problem will have Hopf bifurcations of different criticalities, so extra care should be taken in the analysis of the model.

It is worth mentioning that there is a fourth type of Hopf bifurcation observed in singularly perturbed systems. In this fourth case, there is a Hopf bifurcation in the layer problem but the full system does not possess a Hopf bifurcation. This Hopf bifurcation in the layer problem may cause a *delayed loss of stability *in the full system [39]. A classical example in biophysical models where such a delayed loss of stability plays an important role is given by elliptic bursting (see, e.g., [29,40]).

A wide variety of biophysical models are of the types that are potentially affected by the problems we have discussed in this article, including HH-type neuronal models and many models of intracellular calcium dynamics. In light of our results, it seems advisable that care be taken when attempting to use either quasi-steady-state reductions or GSPT for the analysis of slow-fast systems with Hopf bifurcations.

## Competing interests

The authors declare that they have no competing interests.

## Endnotes

^a^Identifying such a single separation and grouping the state variables roughly into slow and fast families often is a difficult part of the model analysis. ^b^Note that this manifold also represents the phase space for the slow variables *n *in the other singular limit problem on the slow time scale *τ *= *εt*, the reduced problem. ^c^In fact, there will be a manifold of Hopf bifurcations in the layer problem, one associated with each choice of the (fixed) slow variables. We are concerned only with the Hopf bifurcation of the distinguished equilibrium in the layer problem obtained from taking the branch (*v*(*ν*,*ε*), *m*(*ν*,*ε*), *n*(*ν*,*ε*)) in the limit *ε *→ 0. ^d^Note that the reduction of the gates (*m*, *n*) or (*h*, *n*) would remove the Hopf bifurcation from the model. ^e^It should be mentioned that the widely used three-dimensional model captures the most important dynamical feature of pancreatic *β*-cells, namely their bursting behaviour. ^f^It is possible to rescale (locally) slow-fast systems with a singular Hopf bifurcation into a slow-fast system which possesses a Hopf bifurcation in the singular limit, but this Hopf bifurcation of the 'new' layer problem is degenerate [5,36,37]. Hence one also cannot conclude the criticality of the Hopf bifurcation from this singular limit. ^g^Interestingly enough, Fenichel's results [2] were only concerned with the persistence of periodic orbits in the slow manifold but not with that of a Hopf bifurcation.

## Appendix

### Parameter and function definitions

Tables 2 and 3 show the values and function definitions that were used in numerical integration of the HH model, Equations (9) in Section 2.1 and the Chay-Keizer model, Equations (15) in Section 3.2.1, respectively.

**Table 2:** Parameter values and function definitions for the HH model, Equations (9)

${Ē}_{Na}=0.5$	${Ē}_{K}=-0.77$	${Ē}_{L}=-0.544$	${ḡ}_{k}=0.3$	${ḡ}_{l}=0.0025$
*k_v _*= 100 mV	*ε *= 0.0083	*τ_m _*= 1	*τ_n _*= 1	*τ_h _*= 1
$a_{n}\left(v\right)=\frac{0.01\left(k_{v}v+55\right)}{1-exp\left(-\frac{k_{v}v+55}{10}\right)}$	$a_{m}\left(v\right)=\frac{0.1\left(k_{v}v+40\right)}{1-exp\left(-\frac{k_{v}v+40}{10}\right)}$			$a_{h}\left(v\right)=0.07\exp\left(\frac{-k_{v}v-65}{20}\right)$
$b_{n}\left(v\right)=0.125\text{exp}\left(\frac{-k_{v}v-65}{80}\right)$	$b_{m}\left(v\right)=4\text{exp}\left(\frac{-k_{v}v-65}{18}\right)$			$b_{h}\left(v\right)=\frac{1}{exp\left(\frac{-k_{v}v-35}{10}\right)+1}$
$n_{\infty }\left(v\right)=\frac{a_{n}\left(v\right)}{a_{n}\left(v\right)+b_{n}\left(v\right)}$	$m_{\infty }\left(v\right)=\frac{a_{m}\left(v\right)}{a_{m}\left(v\right)+b_{m}\left(v\right)}$			$h_{\infty }\left(v\right)=\frac{a_{h}\left(v\right)}{a_{h}\left(v\right)+b_{h}\left(v\right)}$
$t_{n}\left(v\right)=\frac{1}{a_{n}\left(v\right)+b_{n}\left(v\right)}$	$t_{m}\left(v\right)=\frac{1}{a_{m}\left(v\right)+b_{m}\left(v\right)}$			$t_{h}\left(v\right)=\frac{1}{a_{h}\left(v\right)+b_{h}\left(v\right)}$

**Table 3:** Parameter values and function definitions for the Chay-Keizer model, Equations (15)

*C_m _*= 1*μ*F/cm^2^	${ḡ}_{K,Ca}=0.09\;mS∕cm^{2}$	${ḡ}_{K}=12\text{mS}∕\text{c}{\text{m}}^{2}$
${ḡ}_{\text{Ca}}=6.5\;\text{mS}∕\text{c}{\text{m}}^{2}$	${ḡ}_{L}=0.04\;\text{mS}∕\text{c}{\text{m}}^{2}$	*V_K _*= -75 mV
*V*_Ca _= 100 mV	*V*_L _= -40 mV	V* = 30 mV
*V*' = 50 mV	*K_d _*= 1*μ*M	*f *= 0.004
*k*_1 _= 0.0275 *μ*Mcm^2 ^/nC	*k_c _*= 0.02 ms ^- 1^	
$a_{n}\left(V+V^{*}\right)=0.01\left(\frac{10-V-V^{*}}{exp\left(\frac{10-V-V^{*}}{10}\right)-1}\right)$	$b_{n}\left(V+V^{*}\right)=0.125\;exp\left(\frac{-V-V^{*}}{80}\right)$	
$a_{m}\left(V+V^{\prime }\right)=0.1\left(\frac{25-V-V^{\prime }}{exp\left(\frac{25-V-V^{\prime }}{10}\right)-1}\right)$	$b_{m}\left(V+V^{\prime }\right)=4\;exp\left(\frac{-V-V^{\prime }}{18}\right)$	
$a_{h}\left(V+V^{\prime }\right)=0.07\;exp\left(\frac{-V-V^{\prime }}{20}\right)$	$b_{h}\left(V+V^{\prime }\right)=\frac{1}{exp\left(\frac{30-V-V^{\prime }}{10}\right)+1}$	

## References

[B1] Keener J Sneyd J Mathematical Physiology 2008 2 New York: Springer-Verlag

[B2] Fenichel N Geometric singular perturbation theory J Diff Equ 1979 31 53 98 10.1016/0022-0396(79)90152-9

[B3] Jones CKRT Johnson R Geometric singular perturbation theory Dynamical Systems (Mon-tecatini Terme, 1994). Lecture Notes in Mathematics 1995 New York: Springer

[B4] Krupa M Szmolyan P Extending geometric singular perturbation theory to non-hyperbolic points-fold and canard points in two dimensions SIAM J Math Anal 2001 33 286 314 10.1137/S0036141099360919

[B5] Krupa M Szmolyan P Relaxation oscillation and canard explosion J Diff Equ 2001 174 312 368 10.1006/jdeq.2000.3929

[B6] Szmolyan P Wechselberger M Canards in ℝ^3^ J Diff Equ 2001 177 419 453 10.1006/jdeq.2001.4001

[B7] Szmolyan P Wechselberger M Relaxation oscillations in ℝ^3^ J Diff Equ 2004 200 69 104 10.1016/j.jde.2003.09.010

[B8] Wechselberger M Existence and bifurcation of canards in ℝ^3 ^in the case of a folded node SIAM J Appl Dyn Syst 2005 4 101 139 10.1137/030601995

[B9] Wechselberger M Á propos de canards (apropos canards) Trans. AMS in press

[B10] Ermentrout B Terman D Mathematical Foundation of Neuroscience 2010 New York: Springer-Verlag

[B11] Rubin J Terman D Fiedler B Geometric singular perturbation analysis of neuronal dynamics Handbook of Dynamical Systems 2002 2 Amsterdam: Elsevier Science

[B12] Desroches M Guckenheimer J Kuehn C Krauskopf B Osinga HM Wechselberger M Mixed-mode oscillations with multiple time-scales SIAM Rev in press

[B13] Hodgkin A Huxley A A quantitative description of membrane current and its application to conduction and excitation in nerve J Physiol (Lond) 1952 117 500 544 12991237 10.1113/jphysiol.1952.sp004764 PMC1392413

[B14] FitzHugh R Thresholds and plateaus in the Hodgkin-Huxley nerve equations J Gen Physiol 1960 43 867 896 10.1085/jgp.43.5.867 13823315 PMC2195039

[B15] FitzHugh R Impulses and physiological states in theoretical models of nerve membrane Biophys J 1961 1 445 466 10.1016/S0006-3495(61)86902-6 19431309 PMC1366333

[B16] Nagumo JS Arimoto S Yoshizawa S An active pulse transmission line stimulating nerve axon Proc IRE 1962 50 2061 2070

[B17] Rinzel J On repetitive activity in nerve Feder Proc 1978 37 2793 2802 720633

[B18] Rinzel J Excitation dynamics: insights from simplified membrane models Feder Proc 1985 44 2944 2946 2415401

[B19] Rubin J Wechselberger M Giant Squid-Hidden Canard: the 3D geometry of the Hodgkin Huxley model Biol Cybern 2007 97 5 32 10.1007/s00422-007-0153-5 17458557

[B20] Rinzel J Miller R Numerical calculation of stable and unstable periodic solutions to the Hodgkin-Huxley equations Math Biosci 1980 49 27 59 10.1016/0025-5564(80)90109-1

[B21] Guckenheimer J Oliva R Chaos in the Hodgkin-Huxley model SIAM J Appl Dyn Syst 2002 1 105 114 10.1137/S1111111101394040

[B22] Guckenheimer J Holmes P Nonlinear Oscillations, Dynamical Systems, and Bifurcation of Vector Fields 1983 New York: Springer-Verlag

[B23] Kuznetsov YA Elements of Applied Bifurcation Theory 1998 New York: Springer-Verlag

[B24] Rubin J Wechselberger M The selection of mixed-mode oscillations in a Hodgkin-Huxley model with multiple timescales Chaos 2008 18 015105 10.1063/1.2789564 18377086

[B25] Kuznetsov YA The Andronov-Hopf bifurcation Scholarpedia 2006 1 1858 10.4249/scholarpedia.1858

[B26] Marsden JE McCracken M The Hopf Bifurcation and Its Applications 1976 New York: Springer-Verlag

[B27] Chay TR Keizer J Minimal model for membrane oscillations in the pancreatic *β*-cell Biophys J 1983 42 181 190 10.1016/S0006-3495(83)84384-7 6305437 PMC1329221

[B28] Doedel EJ AUTO-07P: continuation and bifurcation software for ordinary differential equations 2007 http://indy.cs.concordia.ca/auto

[B29] Rinzel J Lee YS Othmer HG On different mechanisms for membrane potential bursting Nonlinear Oscillations in Biology and Chemistry, Lecture Notes in Biomathematics 1986 66 New York: Springer-Verlag

[B30] Atri A Amundsen J Clapham D Sneyd J A single-pool model for intracellular calcium oscillations and waves in the *Xenopus laevis *oocyte Biophys J 1993 65 1727 1739 10.1016/S0006-3495(93)81191-3 8274661 PMC1225900

[B31] Domijan M Murray R Sneyd J Dynamical probing of the mechanisms underlying calcium oscillations J Nonlinear Sci 2006 16 483 506 10.1007/s00332-005-0744-z

[B32] Harvey E Kirk V Osinga HM Sneyd J Wechselberger M Understanding anomalous delays in a model of intracellular calcium dynamics Chaos 2010 20 045104 10.1063/1.3523264 21198116

[B33] Harvey E Kirk V Sneyd J Wechselberger M Multiple time-scales, mixed mode oscillations and canards in intracellular calcium models J Nonlinear Sci

[B34] Baer SM Erneux T Singular Hopf bifurcation to relaxation oscillations SIAM J Appl Math 1986 46 721 739 10.1137/0146047

[B35] Braaksma B Singular Hopf bifurcation in systems with fast and slow variables J Nonlinear Sci 1998 8 457 490 10.1007/s003329900058

[B36] Guckenheimer J Singular Hopf bifurcation in systems with two slow variables SIAM J Appl Dyn Syst 2008 7 1335 1377

[B37] Krupa M Wechselberger M Local analysis near a folded saddle-node singularity J Diff Equ 2010 248 2841 2888 10.1016/j.jde.2010.02.006

[B38] Guckenheimer J Osinga HM The singular limit of a Hopf bifurcation 2011 (preprint)

[B39] Neishtadt A Asymptotic investigation of the loss of stability as a pair of eigenvalues slowly cross the imaginary axis Usp mat Nauk 1985 40 190 191

[B40] Izhikevich E Subcritical elliptic bursting of Bautin type SIAM J Appl Math 2000 60 503 535 10.1137/S003613999833263X

